# Antiretroviral Treatment with Efavirenz Disrupts the Blood-Brain Barrier Integrity and Increases Stroke Severity

**DOI:** 10.1038/srep39738

**Published:** 2016-12-23

**Authors:** Luc Bertrand, Levi Dygert, Michal Toborek

**Affiliations:** 1 University of Miami Miller School of Medicine, Department of Biochemistry and Molecular Biology, Miami, FL, USA

## Abstract

The introduction of antiretroviral drugs (ARVd) changed the prognosis of HIV infection from a deadly disease to a chronic disease. However, even with undetectable viral loads, patients still develop a wide range of pathologies, including cerebrovascular complications and stroke. It is hypothesized that toxic side effects of ARVd may contribute to these effects. To address this notion, we evaluated the impact of several non-nucleoside reverse transcriptase inhibitors (NNRTI; Efavirenz, Etravirine, Rilpivirine and Nevirapine) on the integrity of the blood-brain barrier, and their impact on severity of stroke. Among studied drugs, Efavirenz, but not other NNRTIs, altered claudin-5 expression, increased endothelial permeability, and disrupted the blood-brain barrier integrity. Importantly, Efavirenz exposure increased the severity of stroke in a model of middle cerebral artery occlusion in mice. Taken together, these results indicate that selected ARVd can exacerbate HIV-associated cerebrovascular pathology. Therefore, careful consideration should be taken when choosing an anti-retroviral therapy regimen.

The introduction of anti-retroviral drugs (ARVd) changed the prognostic of HIV infection from a terminal to a chronic disease. Patients can lead normal lives and control the infection by adhering to highly active antiretroviral therapy (HAART)^1^,^2^. Nevertheless, these patients will likely be under treatment for the rest of their lives, meaning that they will be continually exposed to ARVd. Given the length of exposure, infected patients are at higher risk of developing co-morbidities, such as cardiovascular, metabolic and neurological disease^3^,^4^,^5^,^6^,^7^. While some of these disorders can be attributed to HIV infection and the ensuing persistent state of inflammation, growing evidence indicates that ARVd toxicity could be at least partially involved in the development of these pathologies. Several side effects, such as lactic acidosis, lipodystrophy, hyperbilirubinemia, diarrhea, pancreatitis, peripheral neuropathy, neuropsychiatric disorders and hypersensitivity, have been linked to the use of ARVd^8^,^9^,^10^,^11^,^12^,^13^,^14^,^15^,^16^,^17^,^18^. HIV protease inhibitors have been associated with proteasome disruption, liver injury, and gut barrier dysfunction^19^. Non-nucleoside reverse transcriptase inhibitors (NNRTIs) may be responsible for induction of inflammation^20^, neurotoxicity, and rashes^21^. While the direct cause of these adverse reactions can be multifactorial, certain NNRTIs, especially Efavirenz, can play an important role given the indications that they can disrupt nitric oxide production, mitochondrial function, ER stress and autophagy^22^,^23^,^24^,^25^.

The long term survival of HIV infected patients makes them more susceptible to the development of cardiovascular diseases^26^. Several epidemiologic studies denoted that these individuals are younger and present a higher incidence of vasculopathies, cardio- and cerebrovascular diseases than non-infected patients^27^,^28^,^29^,^30^. HIV-positive patients who develop stroke often present a different profile than the general population, exhibiting less predisposing symptoms, such as hypertension^31^. Multiple factors linked to the HIV infection can contribute to this increased susceptibility, including chronic vascular inflammation, opportunistic infections, endocarditis, cachexia, coagulation abnormalities, and dyslipidemia. Furthermore, HIV and its proteins can interact directly with the endothelium and contribute to increased incidence of atherosclerosis, a major contributing factor of cardiovascular disease^32^,^33^, which can further be exacerbated by antiretroviral treatment. Epidemiological studies demonstrated that while endothelial dysfunction is reduced following HAART initiation, long term exposure to ARVd leads to the development of vasculopathy, especially with the usage of certain protease inhibitors^34^,^35^. These conditions could arise from chronic induction of ER stress^36^,^37^, an increase in local inflammation^38^, or apoptosis activation^39^.

The blood-brain barrier (BBB) plays a central role in maintaining the homeostasis of the central nervous system (CNS). The BBB protects the brain from toxins, pathogens, and other potential harmful components that can be present in the blood^40^,^41^,^42^,^43^. The BBB is composed of the neurovascular units in which neurons interact with three major cell types: endothelial cells, pericytes, and astrocytes. The brain microvascular endothelial cells (BMEC) are linked together by an assembly of tight junction (TJ) proteins that restrict the passage of molecules at the paracellular space. The main component of these structures are claudins (primarily claudin-5), which are transmembrane proteins that connect the neighboring endothelial cells. These proteins are linked to the cytoskeleton by adaptor proteins, such as zona occludens-1, 2, 3 and cingulin. In addition, several other molecules also play a role in TJ assembly, such as occludin, junctional adhesion molecules, and adherens junction proteins^40^,^44^. The regulation of TJs is influenced by the signaling factors originating from BMEC, but also influenced by pericytes and astrocytes^40^,^45^,^46^. Dysfunction of the BBB and the associated increase in BBB permeability is linked to the pathology of several acute and chronic CNS diseases. An uncontrolled trafficking of immune cells to the CNS can lead to inflammation, neuronal loss, and the entry of pathogens, resulting in infections. The loss of control on the flow of molecules in or out of the CNS can contribute to the development of Alzheimer’s disease, epilepsy, or stroke^47^. In the context of the present study, it is important that disrupted BBB can increase the extent of tissue damage and mortality in stroke^48^,^49^,^50^.

Several publications reported that ER stress induction is linked to endothelial dysfunction and can lead to an increased vascular permeability in various disease models^51^,^52^,^53^,^54^,^55^,^56^. We previously demonstrated that exposure of brain endothelial cells to Efavirenz induces ER stress and disrupts autophagy^25^. The present study aims to identify the impact of NNRTI-induced toxicity on endothelial functions, such as disruption the barrier integrity and the severity of stroke. Our results demonstrate that of the several NNRTIs tested, only Efavirenz affected endothelial integrity via reduction in claudin-5 expression, and alterations in its phosphorylation. In addition, treatment with Efavirenz resulted in an increase in BBB permeability and enhanced tissue injury in stroke.

## Results

### Exposure to Efavirenz, but not to other NNRTIs, leads to an increase in endothelial permeability

**Figure 1:**
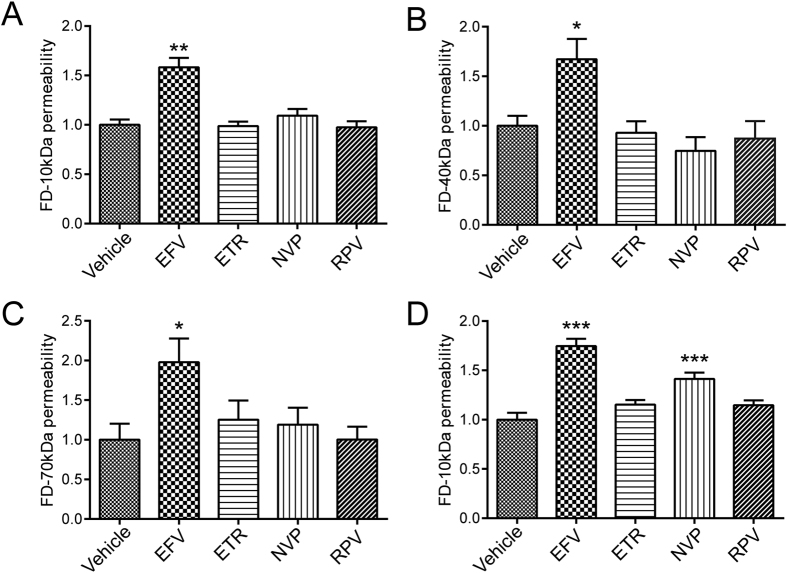
Impact of NNRTIs on endothelial permeability. hCMEC (**A–C**) and human primary brain endothelial cells (**D**), grown to confluence on Transwells, were incubated with vehicle (DMSO), Efavirenz (Efa), Etravirine (ETR), Nevirapine (NVP) or Rilpivirine (RPV) for 48 h. Culture media in the apical compartment of the Transwell system was replaced with medium containing fluorescently tagged dextran (FD) of 10 kDa (**A** and **D**), 40 kDa (**B**) or 70 kDa (**C**). Basolateral levels of FD were assayed 90 min post exposure. Data are mean ± SEM, expressed as fold increase over vehicle control; three independent experiments, each with n = 4; *p < 0.05 vs. Vehicle; **p < 0.01 vs. Vehicle; ***p < 0.001 vs. Vehicle.

To evaluate the impact of NNRTI on endothelial permeability, human cerebral microvascular cells (hCMEC) were grown to confluence on Transwell inserts. After the formation of monolayers, the cells were exposed to the indicated conditions for 48 h. Then, media in the apical compartment of the Transwell system was replaced with medium containing fluorescently-tagged dextran. After 90 min incubation, fluorescence intensity in the basal compartment was read using a plate reader. Among studied NNRTIs, exposure to Efavirenz resulted in a significant increase in permeability of hCMEC monolayers for all molecular sizes of dextran tested (Fig. 1A–C). Exposure to the other NNRTIs did not affect the transfer of fluorescent markers across the endothelial monolayer. These results were largely confirmed in cultures of human primary brain endothelial cells (Fig. 1D). However, exposure to Nevirapine slightly increased permeability across the monolayers of primary brain endothelial cells. Such results appear to be consistent with different susceptibly of primary cells compared to a cell line.

### Efavirenz, but not other NNRTIs, induces a specific reduction in claudin-5 levels

**Figure 2:**
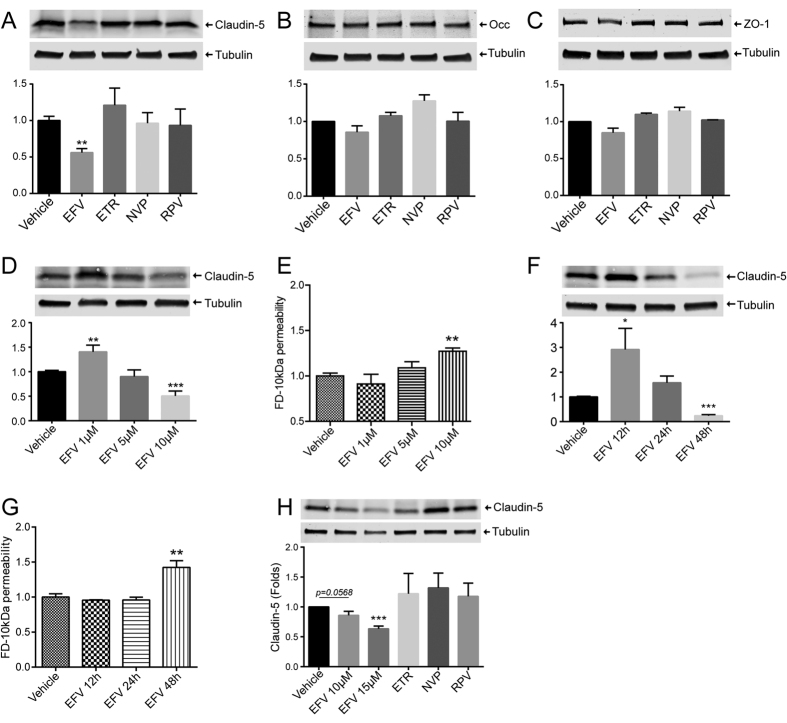
Impact of NNRTIs on tight junction protein expression. hCMEC (**A–G**) and human primary brain endothelial cells (**H**) were grown to confluence, followed by exposure to vehicle (DMSO), Efavirenz (Efa), Etravirine (ETR), Nevirapine (NVP) or Rilpivirine (RPV) for 48 h. Expression of claudin-5 (**A,D,F,H**), occludin (**B**), and ZO-1 (**C**) was assessed by western blotting. Dose-dependent effects of Efavirenz on claudin-5 levels (**D,H**) and endothelial permeability (**E**). Time-dependent impact of Efavirenz on claudin-5 levels (**F**) and endothelial permeability (**G**). Presented blots are cropped from the originals, full size blots available in Supplementary Fig. S1. Data are mean ± SEM, expressed as fold increase over vehicle control; three or four independent experiments, each with n = 4. *p < 0.05 vs. Vehicle; **p < 0.01 vs. Vehicle; ***p < 0.001 vs. Vehicle.

We next sought to identify the impact of NNRTI exposure on TJ protein levels, given their primordial role in maintaining endothelial barrier function. Cells were exposed to NNRTIs as in Fig. 1 and lysed using RIPA buffer. The expression levels of claudin-5, occludin, and ZO-1, three major TJ proteins, were analyzed by western blotting. A specific reduction in claudin-5 levels was observed in cells exposed to Efavirenz, but not to other NNRTIs (Fig. 2A). On the other hand, occludin and ZO-1 levels remained unaffected, independent of treatment (Fig. 2B and C, respectively). In order to evaluate dose response effects of Efavirenz, we exposed hCMEC to doses ranging from 1 μM to 10 μM. Efavirenz at 10 μM consistently lowered claudin-5 levels; however, treatment with 1 μM resulted in a significant increase in expression of this protein (Fig. 2D). No significant impact of Efavirenz on endothelial permeability was observed at doses lower than 10 μM (Fig. 2E).

We also investigated time-dependent effects of Efavirenz exposure on claudin-5 expression and endothelial barrier function. A significant decrease in claudin-5 and endothelial barrier function was confirmed at 48 h of exposure (Fig. 2F and G, respectively). A 12 h treatment with Efavirenz increased claudin-5 levels (Fig. 2F) with no impact on permeability (Fig. 2G). No changes in these parameters were observed in cells exposed to Efavirenz for 24 h.

In human primary brain endothelial cells, exposure to Efavirenz, but not other NNRTIs, lead to a decrease in claudin-5 expression (Fig. 2H). Furthermore, all listed treatments did not impact either occludin or ZO-1 (data not shown), fully confirming the results from a cell line.

Overall, these results indicate that Efavirenz exposure results in an increase in monolayer permeability that correlates with a specific reduction in claudin-5 level. In addition, this effect is contingent on dose and exposure time.

### Disruption of intracellular localization of claudin-5 by Efavirenz

**Figure 3:**
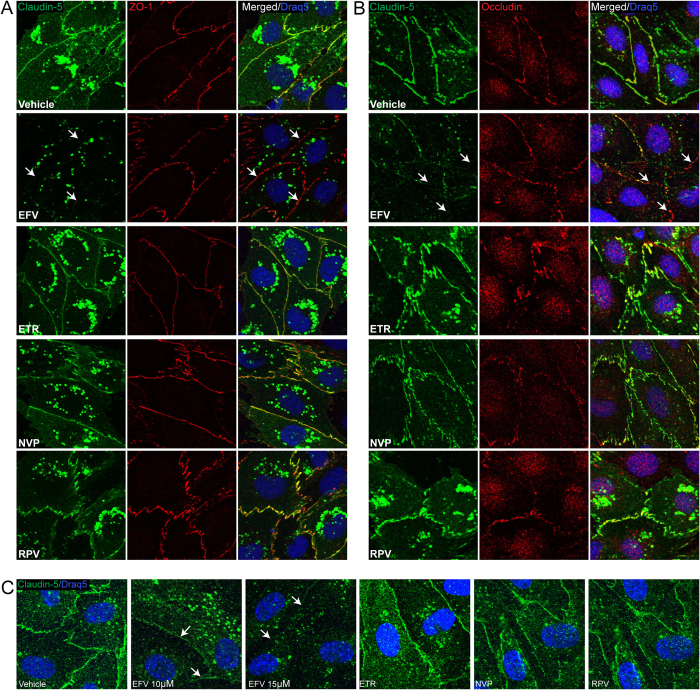
Treatment with Efavirenz, but not with other NNRTIs, decreases claudin-5 immunoreactivity and localization. hCMEC were grown on coverslips to confluence, exposed to NNRTIs as in Fig. 1, and immunostained for claudin-5 (green), ZO-1 (red), or occludin (red). Draq5 (blue) visualizes nuclei. Left and middle columns in **A** and **B** are staining for specific tight junction proteins. The right column in (**A**) is colocalization of claudin-5 with ZO-1, and the right column in (**B**) is colocalization of claudin-5 with occludin. (**C**) Localization of claudin-5 in primary human brain endothelial cells. Arrows indicate discontinuous, absence of claudin-5 immunoreactivity and lack of ZO-1 or Occludin colocalization. EFV: Efavirenz; ETR: Etravirine; NVP: Nevirapine; RPV: Rilpivirine.

In addition to the expression level, cellular localization of TJ proteins is essential for their function. Therefore, we assessed the impact of NNRTIs on claudin-5, occludin, and ZO-1 immunoreactivity. hCMEC were grown to confluence on round coverslips coated with collagen, and exposed to vehicle or NNRTIs for 48 h. Afterward, cells were processed for immunofluorescence to assess cellular localization of TJ proteins. The nuclei were stained with DRAQ5. Using confocal microscopy, we detected a regular colocalization of claudin-5 with ZO-1, and claudin-5 with occludin in cells treated with DMSO, Etravirine, Nevirapine and Rilpivirine (Fig. 3A and B). In contrast, in cells exposed to Efavirenz, this co-localization was absent due to both a severe reduction in the presence of claudin-5 from cell-cell junctions and a general reduction in expression level of this protein (Fig. 3A and B; arrows). Confirming these results, Efavirenz at 10 μM and 15 μM (but not other NNRTIs) decreased claudin-5 immunoreactivity and its presence at cell-cell junctions in primary human brain endothelial cells (Fig. 3C). These results demonstrate that in addition to a reduced expression, Efavirenz treatment affects subcellular localization of claudin-5 at TJs.

### ER stress inhibition protects against Efavirenz-induced a decrease in claudin-5 expression, but not endothelial dysfunction

**Figure 4:**
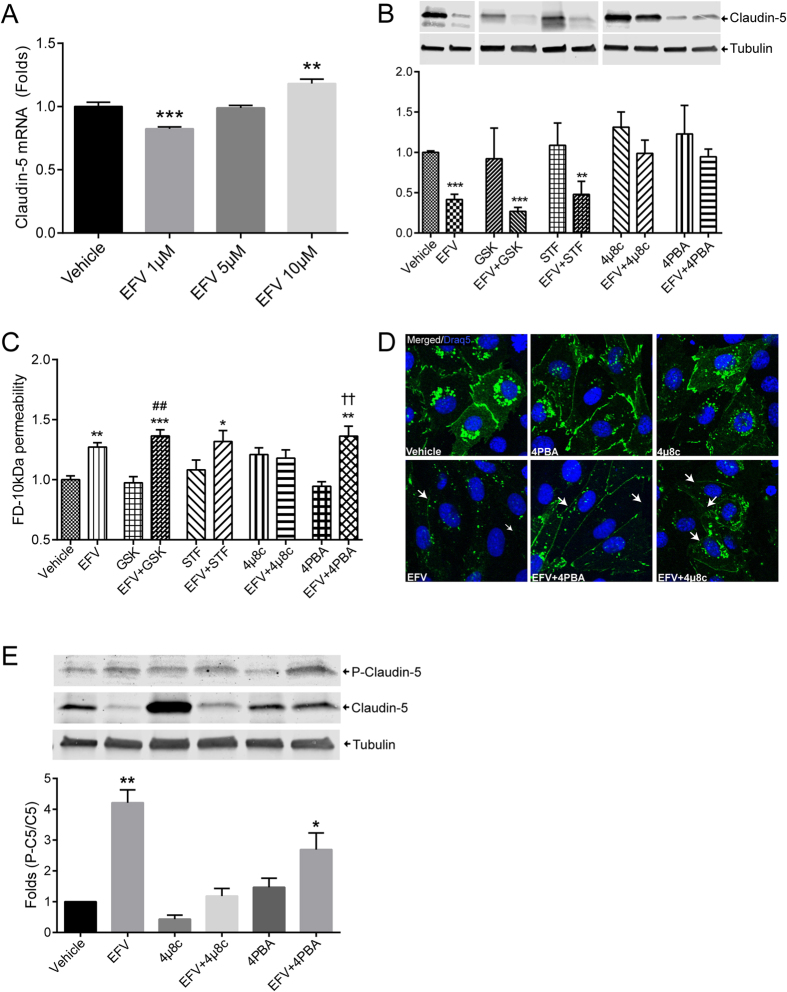
Inhibition of ER stress restores Efavirenz-induced alterations of claudin-5 protein levels but not claudin-5 phosphorylation and disrupted endothelial permeability. (**A**) Dose-depended effects of Efavirenz on claudin-5 mRNA levels as analyzed by real-time PCR. (**B**) Cells were pretreated with various ER stress inhibitors for 6 h before a 48 h exposure to 10 μM Efavirenz, followed by immunoblotting for claudin-5 protein expression. Tubulin was assessed as house-keeping reference. (**C**) Cells were treated as in (**B**) and endothelial permeability was evaluated using FD-10 kDa dye transfer as in Fig. 1. (**D**) Cells were pretreated with selected inhibitors of ER, followed by exposure to Efavirenz as in (**C**), and followed by staining for claudin-5 immunoreactivity (green). Draq5 (blue) visualizes nuclei. Arrows indicate discontinuous or absence of claudin-5 immunoreactivity. (**E**) Claudin-5 phosphorylation levels in cells exposed as in (**D**). Graphs represent ratio of phosphorylated claudin-5 (P-Claudin-5) compared to total claudin-5 levels. Presented blots are cropped from the originals, full size blots available in Supplementary Fig. S1. Data are mean ± SEM, expressed as fold increase over vehicle control; three or four independent experiments, each with n = 4. GSK: GSK2606414; STF: STF-083010; 4PBA: 4-Phenylbutyrate; EFV: Efavirenz. *p < 0.05 vs. Vehicle; **p < 0.01 vs. Vehicle; ***p < 0.001 vs. Vehicle; ^##^p < 0.01 vs. GSK; ^††^p < 0.01 vs. 4PBA.

To identify the mechanisms of Efavirenz-induced claudin-5 downregulation, we first evaluate mRNA levels in cells exposed to increasing doses of this drug. While small changes in claudin-5 mRNA were detected (Fig. 4A), they did not correlate with the observed changes at the protein level. Specifically, a decrease in claudin-5 mRNA was observed in cells exposed to Efavirenz at 1 μM, i.e., the levels of the drug which increased claudin-5 protein level. In addition, 10 μM Efavirenz elevated claudin-5 mRNA (Fig. 4A); however, the same dose of Efavirenz consistently decreased claudin-5 protein expression (Figs 2 and 3). These results suggested a post-transcriptional mechanism of Efavirenz-induced alterations of claudin-5 expression.

We previously demonstrated that Efavirenz can induce ER stress^25^, which is a cellular response that can affect protein expression, via at least two ER sensors: PERK and IRE1α. Therefore, we evaluated whether induction of ER stress is involved in Efavirenz-induced alterations of claudin-5 expression. hCMEC were pretreated with several ER stress inhibitors (GSK2606414, PERK inhibitor; STF-083010, IRE1α inhibitor; 4 μ8c, IRE1α inhibitor; or 4-phenylbutyrate [4PBA] a broad inhibitor/chaperone) for 6 h, followed by treatment with Efavirenz for 48 h. As indicated in Fig. 4B, claudin-5 expression was restored to the control levels by 4 μ8c and 4PBA. However, treatment with 4 μ8c disrupted endothelial barrier function, and 4PBA was not effective in protecting against Efavirenz-induced disruption of endothelial permeability (Fig. 4C). These results indicate that ER stress inhibition can restore protein levels of claudin-5; however, this recovery does not fully extend to endothelial integrity. To explain this discrepancy, we observed that pretreatment with 4 μ8c or 4PBA failed to restore claudin-5 staining at cell junctions (Fig. 4D, arrows).

TJ protein levels are critical for TJ assembly and the barrier function, however an additional factor that may affect endothelial permeability is their phosphorylation. It has been demonstrated that claudin-5 phosphorylation results in an increase in BBB permeability, and that this modification can be in part mediated by Rho kinases^57^. Therefore, we analyzed phosphorylation levels of claudin-5 by western blotting in cells exposed to Efavirenz. The results indicated that Efavirenz resulted in a significant increase in claudin-5 phosphorylation, which was not restored by pretreatment with 4 μ8c or 4PBA (Fig. 4E). This indicated that while 4PBA prevents Efavirenz mediated reduction in claudin-5 proteins levels, phosphorylation levels remain high, the effect that correspond with disrupted barrier function.

### Treatment with Efavirenz, but not other NNRTIs, disrupts TJs in an animal model

**Figure 5:**
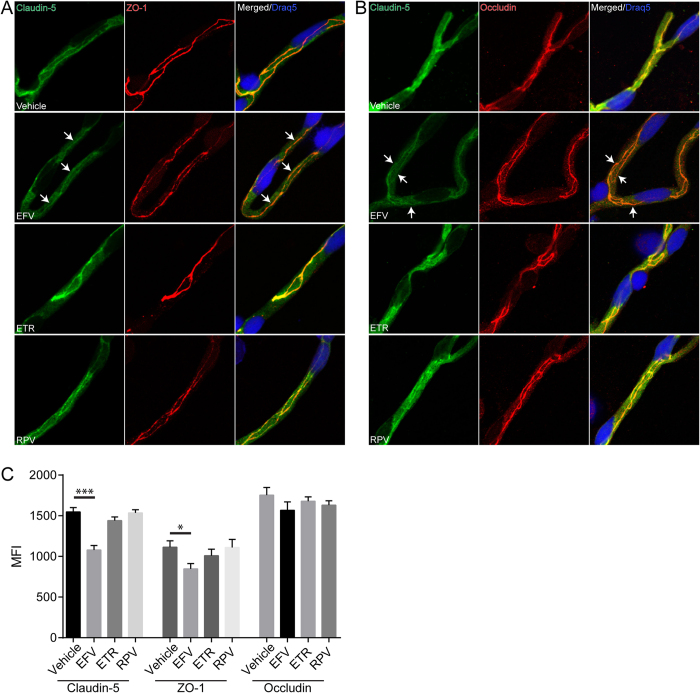
Treatment with Efavirenz, but not other NNRTIs, affects claudin-5 immunoreactivity and localization in brain microvessels. Mice were treated with various NNRTIs or vehicle for 30 days as described in the Materials and Methods. Claudin-5 (green), ZO-1 (red), and occludin (red) were analyzed by immunostaining in isolated brain microvessels. Nuclei were stained with Draq5 (blue). Left and middle columns in **A** and **B** are staining for specific tight junction proteins. The right column in (**A**) is colocalization of claudin-5 with ZO-1 and the right column in (**B**) is colocalization of claudin-5 with occludin. Arrows indicate discontinuous, absence of claudin-5 immunoreactivity and lack of ZO-1 or Occludin colocalization. (**C**) Quantification of mean fluorescence index (MFI) signal for tight junction proteins analyzed in isolated microvessels. Data obtained from 3 mice per group, 5–7 microvessels per mice. *p < 0.05; ***p < 0.001. EFV: Efavirenz; ETR: Etravirine; RPV: Rilpivirine.

Following the *in vitro* analyses, we next evaluated whether Efavirenz-induced alterations of TJ proteins occur *in vivo* in an animal model. Mice were gavaged with either vehicle, Efavirenz, Etravirine, or Rilpivirine for 30 days. Afterward, brains were harvested, the microvessels were isolated and analyzed by immunofluorescence for the expression and localization of claudin-5, occludin, and ZO-1 (Fig. 5A and B). Among all NNRTIs used, only treatment with Efavirenz resulted in reduction of claudin-5 and ZO-1 immunoreactivity. Quantitative analysis of signal intensity by mean fluorescence intensity (MFI) demonstrated a significant decrease in claudin-5 and to a lesser degree in ZO-1 in mice exposed to Efavirenz as (Fig. 5C). These changes were associated with disrupted claudin-5 colocalization with ZO-1 (Fig. 5A) and occludin (Fig. 5B). These results indicate that Efavirenz-induced TJ disruption occurs *in vivo* in a physiologically relevant treatment regimen.

### Efavirenz disrupts TJ integrity in EcoHIV/NDK-infected brains

**Figure 6:**
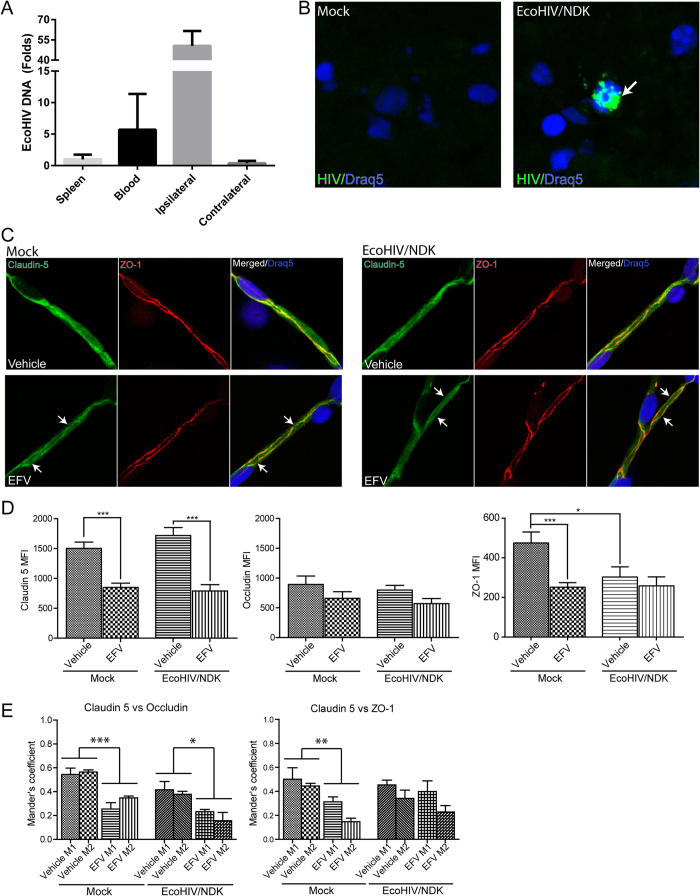
Tight junction protein expression and colocalization in EcoHIV/NDK-infected brains. Mice were infected with the mouse adapted strain EcoHIV/NDK infused into the common carotid artery. (**A**) Real-time PCR analysis of HIV DNA content one week post infection. Bars represent fold changed compared to spleen HIV DNA content (n = 5). (**B**) *In situ* PCR analysis of HIV presence in brain sections of mock or EcoHIV/NDK infected mice. Positive HIV amplification is indicated in green (arrow). Nuclei are stained with Draq5 (blue). (**C**) Immunofluorescence of tight junction proteins in brain microvessels isolated from mock (left panel) or EcoHIV/NDK-infected (right panel) mice and treated with vehicle or Efavirenz (10 mg/kg for 30 days). Slides were immunostained for claudin-5 (green) and ZO-1 (red); Draq5 (blue) was used to stain nuclei. Arrows indicate discontinuous, absence of claudin-5 immunoreactivity and lack of ZO-1 colocalization. (**D**) Mean fluorescence index analysis of tight junction protein staining in isolated brain microvessels as in (**C**). Claudin-5, ZO-1, and occludin immunoreactivity was analyzed. (**E**) Analysis of colocalization of claudin-5 with ZO-1 or claudin-5 with occludin using the Mander’s coefficient. (**C–E**) Data obtained from 5 mice per groups, 5–9 microvessels per mice. Data are mean ± SEM. Analysis was performed using Image J and JACoP plugin. EFV: Efavirenz. *p < 0.05; **p < 0.01; ***p < 0.001.

Given that ARVd are typically used in HIV-infected individuals, we next compared the impact of Efavirenz on TJ integrity in mice infected with a chimeric HIV virus specifically adapted for mice, EcoHIV/NDK. To target the brain for infection, the virus was infused into the internal cerebral artery^58^, mimicking the initial stages of HIV brain infection in which the blood-borne virus enters the brain via the BBB. EcoHIV inoculation resulted in a preferential infection of the ipsilateral hemisphere, which corresponds to the site of virus infusion (Fig. 6A). The infection was confirmed by *in situ* PCR, which detected EcoHIV-infected cells mainly in the striatum (Fig. 6B). Seven days post infection, mice were treated for 30 days with Efavirenz or vehicle. Then, brain microvessels were isolated and immunostained for occludin (images not shown), claudin-5, and ZO-1 (Fig. 6C). The expression values of TJ proteins were calculated as MFI.

The infection with EcoHIV did not alter the MFI values for claudin-5 and occludin; however, ZO-1 levels were diminished as compared to mock infection (Fig. 6C and D). Treatment with Efavirenz markedly decreased claudin-5 levels in both animal groups, did not altered occludin levels, and diminished ZO-1 expression only in mock-infected mice. In addition, a reduction in colocalization of claudin-5 with occludin was identified using the Mander’s coefficient in both mock- and EcoHIV-infected mice (Fig. 6E). Colocalization of claudin-5 with ZO-1 was decreased only in mock-infected mice.

### Efavirenz treatment increases BBB permeability and amplifies stroke severity in HIV-infected brains

**Figure 7:**
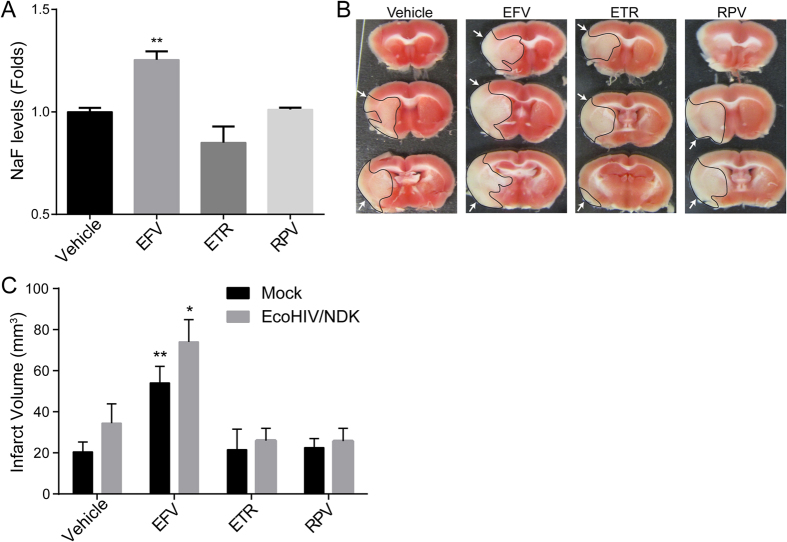
Efavirenz increases BBB permeability and stroke tissue injury. (**A**) Quantification of BBB permeability. Mice were treated for 30 days with either vehicle or NNRTIs as described in the Material and Methods. Translocation of sodium fluorescein (NaF) from plasma into the brain parenchyma was used as the indicator of BBB integrity. Data are mean ± SEM, expressed as fold change compared to vehicle, n = 5 per group. To analyze infarct volume, mice were either mock or EcoHIV/NDK infected and treated as in (**A**). Brains were stained with TTC 24 h after a 60 min occlusion of the middle cerebral artery occlusion (MCAO). (**B**) Representative images of infarct areas in brain slices from NNRTI-treated mock-infected mice. Infarct areas are highlighted and indicated by arrows. (**C**) Quantification of total infarct volume from all animal groups. Data are mean ± SEM, n = 4–7 animals per group. *p < 0.05 vs. Vehicle; **p < 0.01 vs. Vehicle.

Taking into consideration that TJs play a primordial role in maintaining BBB integrity, we evaluated the impact of NNRTIs on the BBB permeability. Animals were treated with NNRTIs as in Fig. 6, followed by intraperitoneal injection with sodium fluorescein (NaF), which was allowed to circulate for 20 min. Mice were then euthanized; blood was collected via cardiac puncture, and animals were perfused with saline. Brains were carefully harvested and processed for NaF quantitation. Treatment with Efavirenz, but not with other NNRTIs, induced a significant increase in NaF levels in the brains, indicating disrupted barrier function of the BBB (Fig. 7A). These results demonstrate that the disruption of TJ integrity caused by Efavirenz has functional implications on BBB permeability.

We next evaluated whether exposure to specific NNRTIs contributes to the development of stroke during HIV infection. HIV-infected patients are known to have a higher risk of stroke, which is often also more severe. Furthermore, stroke development and severity is exacerbated by a disrupted BBB^50^.

Mice were infected with EcoHIV and treated with NNRTIs as in Fig. 6, followed by induction of stroke by the middle cerebral artery occlusion (MCAO) for 60 min and reperfusion for 23 h^59^. Afterward, brains were harvested, sliced, and stained to analyze infarct volume. Among all tested NNRTIs, only Efavirenz treatment induced a significant increase in stroke severity when compared to vehicle. This phenomenon was observed both in mock and EcoHIV-infected mice (Fig. 7B and C). While there was a trend for a larger infarct volume in infected animals, the differences between the groups were below the threshold of statistical significance. These results indicate that while HIV is a potential contributor to stroke, Efavirenz has a highly significant impact on stroke severity, almost doubling infarct volume when compared to vehicle controls.

## Discussion

The suppression of HIV replication using ARVd is essential in maintaining the health of seropositive patients. However, given that patients require antiretroviral therapy for the rest of their lives, drug toxicity is an important factor to take into consideration to prevent complications, and decrease the potential of exacerbating secondary diseases. Several publications demonstrated that selected ARVd used in the treatment of HIV have serious side effects which can affect patient health outcome. In addition, while HAART can suppress viral replication below detectable levels, there is a continuous presence of circulating viral proteins that alone can contribute to disease and lower the threshold of ARVd toxicity^60^.

While several categories of ARVds exist and are used in HIV-infected patients, we focused on toxicity of NNRTIs, selecting drugs from the first and second design generation that are currently in use in clinics. Even though Efavirenz was recently removed from the initial treatment option in the US, it is still part of the first NNRTI based regimen recommended by the WHO, and remains commonly used worldwide. Second generation NNRTIs have also been included in this study to evaluate cerebrovascular effects and safety of these drugs. We, and others, reported that Efavirenz induces ER stress and affects the autophagy pathway^23^,^24^,^25^, which are likely to affect other pathways and impact cell functions in biological systems. In the current study, we investigated the toxicity of Efavirenz, as well as Etravirine, Nevirapine, and Rilpivirine, by focusing on one of the primary roles of brain endothelial cells, namely the integrity of the BBB.

The structure of the BBB relies on the function of multiple cells, namely endothelial cells, pericytes, and astrocytes. While conditions affecting any of those cells can compromise the integrity of this barrier, we focused our investigation on brain microvascular endothelial cells given their central role in the formation and regulation of the BBB. Our results demonstrate that at physiologically relevant concentrations, of all of the NNRTIs tested, only Efavirenz affected endothelial integrity, leading to an increase in permeability. While we observed a slight increase in permeability of primary brain endothelial cells exposed to Nevirapine, none of the other tests performed confirmed endothelial toxicity of this drug. The barrier function is highly reliant on the formation of TJs, composed especially claudin-5, ZO-1, and occludin, which were included in the present study. Claudin-5 was demonstrated to be an important contributor to BBB integrity^61^ and its upregulation correlates with a protective effect against CNS tissue injury^62^. Similar observations have been made for ZO-1 and occludin both *in vitro* and *in vivo*^62^,^63^,^64^,^65^. In our model system, exposure to Efavirenz selectively decreased the levels of claudin-5. These effects were dose- and time-dependent, and were observed both *in vitro* (in two different model systems) and *in vivo*. Efavirenz also had an impact on intracellular localization of this protein, with the prominent effect on removing claudin-5 from cell-cell junctions. In contrast, no changes in expression levels or localization were observed for occludin in our model systems. ZO-1 levels were not affected *in vitro*; however, they decreased upon Efavirenz administration in an animal model. This observation is important as it demonstrates that *in vitro* drug toxicity assays do not fully reproduce responses in more complex biological systems. Treatment with Efavirenz also disrupted the integrity of the BBB *in vivo*, which is consistent with the role of claudin-5 in maintaining the BBB barrier function^66^.

We next sought to identify the mechanisms of claudin-5 disruption. Given our previous observations that Efavirenz induced ER stress in brain endothelial cells, we investigated the role of this process in alterations of claudin-5 expression and localization. This line of investigation is consistent with the reports on the role of ER stress in vascular dysfunction in coronary disease^67^,^68^, diabetic retinopathy^69^ and, several other processes involving endothelium^70^,^71^. While the role of claudin-5 in maintaining vessel integrity is well established, limited data is available on the impact ER stress on integrity of claudin-5 and other TJ proteins^51^,^72^,^73^,^74^.

We demonstrated previously that the PERK and IRE1α pathways are involved in Efavirenz-induced ER stress. Based on these results, several inhibitors were employed in the present study to explore the link between ER stress, claudin-5, and disrupted endothelial permeability. We identified two inhibitors, namely 4 μ8c and 4PBA, which prevented Efavirenz-induced alterations of claudin-5 expression; however, they did not restore endothelial barrier function. Regarding 4 μ8c, given that it alone resulted in an increase in endothelial permeability, it is reasonable to assume that an off target effect could be at play. On the other hand, 4PBA restored the total levels of claudin-5 in Efavirenz-treated cells; however, immunostaining pattern exhibited a preserved diminished intensity and discontinuity at cell-cell junctions. In addition, 4PBA pretreatment did not restore Efavirenz-induced alterations of claudin-5 phosphorylation, a process that is influenced by Rho kinases and leads to a decrease in TJ tightness^75^,^76^. To support this notion, it has been shown that Efavirenz can increase cytoplasmic calcium levels, which has been linked to Rho kinase activation^77^. In addition, HIV-1 is known to activate RhoA^57^, further contributing to BBB disruption in seropositive patients and potentially increasing neuroinvasion.

In light of the concerns that ARVd can contribute to neurotoxicity in HIV-1 infection, we employed a mouse adapted strain of HIV called EcoHIV/NDK in which the viral gp120 protein was replaced with gp80 for the tropism to mouse cells^78^,^79^,^80^,^81^. To mimic a natural route of infection while promoting CNS entry, virus was inoculated by infusion via the internal cerebral artery. This technique induces a strong preference for CNS infection in the infused hemisphere. Compared to mock infection, we did not observe any differences in claudin-5 expression in either the vehicle- or Efavirenz-treated group. Nevertheless, brain infection with EcoHIV/NDK diminished expression of ZO-1 intensity in brain microvessels. This decreased expression corresponded to the impact of Efavirenz treatment, and no further reduction was observed in the HIV plus Efavirenz group as compared to HIV or Efavirenz alone. The link between ZO-1 and HIV has previously been identified and was associated, at least in part, to the toxic effect of HIV Tat protein^82^,^83^,^84^,^85^.

While several diseases are associated with HIV infection, cerebrovascular events are among the leading causes of mortality^86^,^87^,^88^. Therefore, we also evaluated the impact of NNRTI treatment on stroke in both mock or HIV infected mice. Our novel results indicate that Efavirenz treatment significantly increased stroke severity, inducing a two-fold increase in tissue damage as compared to controls. These results are consistent with the impact of this drug on claudin-5 expression and BBB integrity. HIV infection lead to a small increase in infarct volume in all treatment groups; however, it was below the threshold of significance.

In summary, our findings indicate that while HAART is an integral part of HIV treatment in seropositive patients, the choice of drugs used in the treatment regimen is crucial in preventing toxicity. Indeed, selected NNRTIs, such as Efavirenz, can deteriorate treatment outcome, especially in people subjected to cerebrovascular disease.

## Material and Methods

### Cell culture and virus stock preparation

Human cerebral microvascular endothelial cells (hCMEC; hCMEC/D3 cell line) were obtained by immortalizing human brain microvascular cells^89^. The cells are very well characterized and have been widely used in the literature, including our studies on ARVd toxicity^89^. The cells were cultured in EBM-2 medium (Lonza) supplemented with VEGF, IGF-1, EGF, basic FGF, hydrocortisone, ascorbate, gentamycin, and 0.5% fetal bovine serum (FBS, Lonza) in cell culture dishes coated with rat-tail collagen I (BD Bioscience) in 5% CO_2_ humid incubator at 37 °C. HEK293T/17 cells (ATCC) were cultured in DMEM medium supplemented with 10% FBS. Primary human brain endothelial cells (Cell Systems) were cultured in CSC complete medium on dishes coated with CSC attachment factor (all from Cell Systems).

Chimeric HIV virus EcoHIV/NDK was used for mouse infection. EcoHIV/NDK was generated by replacing the viral gp120 protein with gp80 for the ecotropic moloney murine leukemia virus^78^. The viral plasmid was a kind gift from Dr. David Volsky (Icahn School of Medicine at Mt. Sinai, New York, NY). Viral stocks were prepared by transfecting HEK 293 T/17 cells with the plasmid using lipofectamine 2000 (Invitrogen). Viral concentration in the filtered supernatant was quantified using p24 ELISA kit (Zeptometrix).

### Drugs and inhibitor treatment

Exposure to NNRTIs was conducted in serum-free and antibiotic-free media for 48 h at the following concentrations: Efavirenz (1, 5, 10, and 15 μM), Etravirine (1.6 μM), Rilpivirine (0.5 μM), and Nevirapine (7.8 μM). These concentrations reflect physiological plasma concentrations of the drugs^90^,^91^. Regarding Efavirenz levels, both 10 and 15 μM are relevant to human exposure. While the mean plasma concentration of Efavirenz can vary from 3.17 to 12.67 μM in the majority of patients, a proportion of them (up to 14% in some studies) exhibit even higher concentrations^91^. ARVds were acquired from the NIH AIDS Reagent Program (NIAID, Germantown, MD).

Activation of ER stress was blocked using the following inhibitors: GSK2606414 (7 μM), STF-083010 (10 μM), 4 μ8c (3 μM) (All EMD-Millipore) and 4-phenylbutyrate (2 mM, Sigma-Aldrich). Pre-exposure to ER stress inhibitors was initiated 6 h before NNRTI treatment.

### Animal treatment, infection with EcoHIV/NDK, and the middle cerebral artery occlusion procedure

All animal procedures were approved by the University of Miami Institutional Animal Care and Use Committee in accordance with National Institutes of Health (NIH) guidelines and performed in accordance with the relevant guidelines and regulations. Male C57BL/6 J mice (Jackson Laboratories) were allowed to acclimatize to the animal facility for one week with free access to food and water. They were then infused with EcoHIV/NDK (200 ng of p24) into the internal carotid artery using a method previously described^58^,^92^,^93^. Control animals received saline. Administration of NNRTIs started seven days post infection, and lasted for 30 days. Mice were gavaged daily with either 12.5% DMSO in saline (control), 10 mg/kg Efavirenz, 6.6 mg/kg Etravirine, or 0.42 mg/kg Rilpivirine (NIH AIDS Reagent Program). Afterward, mice were sacrificed for microvessels isolation or were used for *in vivo* BBB permeability assay or stroke studies.

Stroke was induced following the drug administration by the middle cerebral artery occlusion (MCAO) as described^59^,^94^. Briefly, occlusion was performed by insertion a silicone coated suture (Doccol) into the common cerebral artery and blocking blood flow to the middle cerebral artery for 60 min. Afterward, the suture was removed and blood flow restored for 23 h. Brains were harvested, sliced using a 1 mm brain matrix (Braintree Scientific) and stained with 2,3,5-triphenyltetrazolium chloride (TTC, ThermoFisher). The images were captured using a digital camera and infarct volume was analyzed using Image J.

### Permeability assays

*In vitro* endothelial permeability assays were performed in 12 wells, 0.4 μm Transwells plates (Corning) using either hCMEC (seeded at 6 × 10^4^ cells per insert) or human primary brain endothelial cells (seeded at 5 × 10^4^ cells per insert). Medium was changed every 48 h and monolayer formation was monitored using trans-epithelial electrical resistance. Seven days after seeding, medium was changed in the upper chamber for one containing treatment factors. Following exposure, media was replaced with Hanks balanced salt solution in both chambers; however, fluorescently tagged dextran (10, 40 or 70 kDa) was added to the upper chamber at the concentration of 0.5 mg/ml. Fluorescent marker translocation was analyzed after 90 min incubation by transferring 100 μl aliquots from the lower chamber to a 96 well plaque and reading fluorescence at 485 nm (Ex) and 525 nm (Em).

*In vivo* BBB permeability assay was performed using sodium fluorescein (NaF) as described before^95^. Briefly, mice were injected intraperitoneally with 200 μl of 10% NaF solution, which was allowed to circulate for 20 min. Mice were euthanized, blood was collected via heart puncture, and animals were perfused using normal saline. Brain hemispheres were thoroughly homogenized in PBS using a tissuelyser system (Qiagen) and cleared of debris by centrifugation. Sample protein concentration was measured to normalize the results. Proteins were precipitated with 100% TCA (Sigma-Aldrich), followed by centrifugation. Supernatants were mixed with 0.05 M sodium tetraborate buffer and NaF florescence was measured in the supernatants using a fluorescent plate reader (485 nm Ex, and 525 nm, Em). In addition to the brain, plasma NaF levels were assessed to control for injection variation.

### Immunoblotting and immunostaining

Cells were exposed to vehicle or NNRTIs in 100 mm dishes, washed, and lysed in RIPA buffer (Santa Cruz Biochemical) containing protease/phosphatase inhibitors (Cell Signaling) 48 h post-exposure, unless otherwise stated. Protein concentration was assessed using a BCA protein assay (Pierce), and 20 μg of protein per sample was loaded on a TGX 4–20% gradient gel (Biorad). Transfer was performed on a nitrocellulose membrane using a Trans-blot Turbo system (Biorad). Afterward, membranes were blocked in 4% BSA in TBS. Primary antibodies were incubated for 1.5 h at room temperature in blocking buffer supplemented with 0.1% Tween-20 and applied at the following concentrations: 1:1000 (claudin-5, Cell Signaling (Rb) and Invitrogen (Ms); claudin-5 (P-Y217), Abcam; ZO-1, Invitrogen; occludin, Invitrogen) or 1:10,000 (Tubulin, Sigma). Membranes were imaged in a Licor CLX imaging system with the secondary antibodies diluted at 1:20 000 (anti-rabbit 800CW and anti-mouse 680RD, Licor). Signal quantification was performed using Image Studio 4.0 software (Licor).

Immunostaining was performed on cultured cells or isolated microvessels, which were heat-fixed on slides and processed as previously described^25^. Samples were stained with rabbit antibodies against claudin-5 (Abcam) and ZO-1 (Cell Signaling), or with mouse antibodies against claudin-5 (Invitrogen) and occludin (Invitrogen). Alexa-488 and -594 secondary antibodies (Invitrogen) were used for target detection; DRAQ5 (Cell Signaling) was employed to stain cell nuclei. Imaging was performed on an Olympus Fluoview 1200 confocal microscope with a 60x oil immersion lens, and analyzed using ImageJ software.

### HIV detection by *in situ* PCR

*In situ* PCR was used to detect cells infected with HIV. Brains of mice either mock- or EcoHIV/NDK infected were harvested 1 week post infection, flash-frozen in liquid nitrogen, sliced at 10 μm, and kept at −80 °C until analysis. Slides were processed for *in situ* PCR as described^96^. Briefly, sections were fixed in 4% paraformaldehyde for 4 h at room temperature and then digested using protease K at 5 μg/ml for 12 min at 25 °C. The enzyme was inactivated by a 95 °C incubation for 2 min. PCR mixture was prepared as described^96^, but modified for primers labeled with Alexa-594 to directly detect the PCR product. The following primers were used: NDK_F 5′-ALEXA 594-GGACCACAGGCTACACTAGAAG; NDK_R 5′-CAGCCAAAACTCTTGCTTTATG. Slide PCR was performed using an Xmatrx mini (Biogenex) cycler and Frame-seal slide chambers (Biorad). Slides were fixed at 92 °C for 1 min, stained with DRAQ5, and washed twice in 2x saline sodium citrate buffer. Imaging was performed as described in the section on immunofluorescence.

### Real-time PCR

mRNA was isolated using RNeasy mini kit (Qiagen) and reverse transcribed by reverse transcription system (Promega) according to manufacturer’s instructions. Real time PCR was performed using an Applied Biosystem 7500 System and primers and probes for claudin-5, ZO-1 and occludin (ThermoFisher). GAPDH mRNA level was assessed in duplication for data normalization.

Tissue detection of EcoHIV/NDK was performed 1 week post infection. Tissue was harvested and processed using All Prep DNA/RNA isolation kit (Qiagen). RNA was reverse transcribed as described above. HIV detection was then conducted using the following primers and probe: NDKgag_F 5′-GACATAAGACAGGGACCAAAGG; NDKgag_R 5′-CTGGGTTTGCATTTTGGACC; NDKgag_Probe 5′-AACTCTAAGAGCCGAGCAAGCTTCAC. Normalization was conducted based on GAPDH for RNA or mouse HBB for DNA^80^.

## Additional Information

**How to cite this article**: Bertrand, L. *et al*. Antiretroviral Treatment with Efavirenz Disrupts the Blood-Brain Barrier Integrity and Increases Stroke Severity. *Sci. Rep.* **6**, 39738; doi: 10.1038/srep39738 (2016).

**Publisher's note:** Springer Nature remains neutral with regard to jurisdictional claims in published maps and institutional affiliations.

## Supplementary Material

Supplementary Information
