# Supplementary material for: Antiretroviral Treatment with Efavirenz Disrupts the Blood-Brain Barrier Integrity and Increases Stroke Severity
Source: Sci Rep. 2016 Dec 23;6:39738. doi: 10.1038/srep39738 (PMC5180178; doi:10.1038/srep39738)
Supplement: Supplementary Information [file srep39738-s1.pdf]

ANTIRETROVIRAL TREATMENT WITH EFAVIRENZ DISRUPTS THE BLOOD-BRAIN BARRIER INTEGRITY AND INCREASES STROKE SEVERITY

Luc Bertrand, Levi Dygert and Michal Toborek

SUPPLEMENTAL INFORMATION

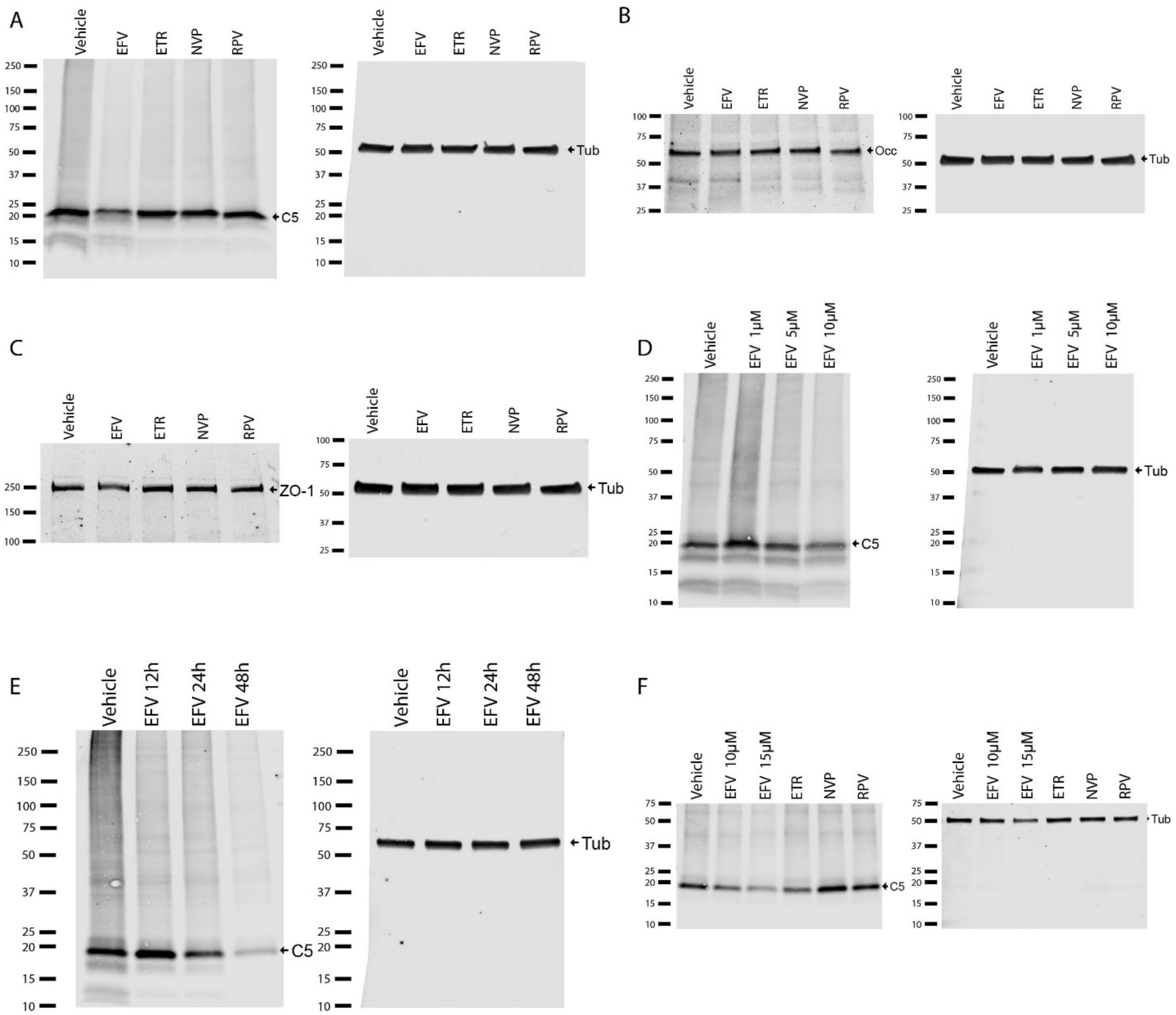

Supplementary Figure S1. Full size blots illustrated in Figure 2. (A) Representative full size blot used in Figure 1A. (B and C) Representative full size blots used in Figures 2B and C, respectively. A single blot was cut at molecular weight marker 100 kDa to allow for simultaneous imaging of occludin and ZO-1. (D) Representative full size blot used in Figure 2D. (E) Representative full size blot used in Figure 2F. (F) Representative full size blot used in Figure 2 H. Abbreviations: C5, claudin-5; EFV, Efavirenz; ETR, Etravirine; NVP, Nevirapine; Occ, occludin; RPV: Rilpivirine; Tub, tubulin.

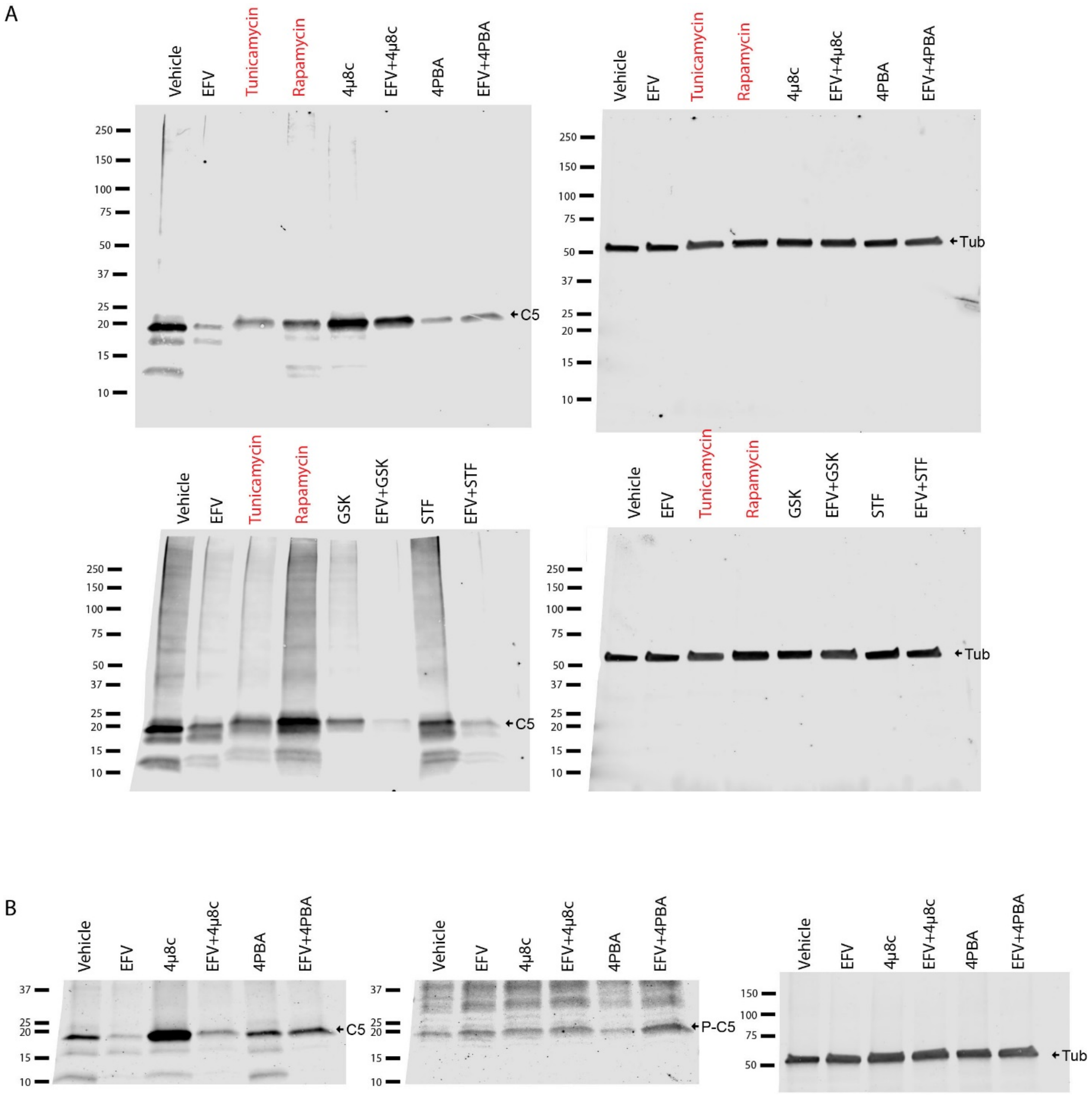

Supplementary Figure S2. Full size blots illustrated in Figure 4. (A) Representative full size blots used in Figure 4B. The final Figure 4B was cropped from two separate western blots illustrated on the upper and lower panels. The same Vehicle and Efavirenz samples were run on both western blots. Treatments indicated in red (Tunicamycin and Rapamycin) were removed from the final submission as they were not the subjects of this study. (B) Representative full size blots used in Figure 4E. The original blot was cut above molecular weight marker 37kDa to allow for triple staining of proteins on the same western blot. Abbreviations: 4PBA, 4-phenylbutyrate; C5, claudin-5; GSK, GSK2606414; p-C5, phosphorylated claudin-5; STF, STF-083010; Tub, tubulin.
